# An update on recent developments in rupture of renal angiomyolipoma

**DOI:** 10.1097/MD.0000000000010497

**Published:** 2018-04-20

**Authors:** Chenyang Wang, Xinyuan Li, Linglong Peng, Xin Gou, Jing Fan

**Affiliations:** a Zhongshan School of Medicine, Sun Yat-Sen University, Guangzhou, Guangdong; b Department of Urology, Chongqing Medical University First Affiliated Hospital, Chongqing, China.

**Keywords:** aneurysm, angiomyolipoma, prognosis, radiology, rupture, treatment

## Abstract

**Background:**

Renal angiomyolipoma (AML) is a common benign tumor of the kidney. The main complication of AML is retroperitoneal hemorrhage caused by AML rupture, which can be severe and life threatening. The risk of AML rupture used to be determined by tumor size. However, these criteria have been challenged by series of clinical studies and case reports, suggesting prediction AML rupture based on tumor size is not always reliable.

**Methods:**

The authors searched PubMed using “angiomyolipoma,” “AML,” and “rupture” and reviewed relevant studies. The authors investigated the risk factors of AML rupture using the retrieved literature. The authors also summarized current modalities to evaluate and manage AML.

**Results:**

It is established that risk of AML rupture is associated with lesion size. However, genetic abnormality, aneurysm formation, and pregnancy are also risk factors for tumor rupture. Thus, the prediction of AML rupture should be based on a more comprehensive risk assessment system. The management of renal AML and tumor rupture was also discussed in the present paper.

**Conclusion:**

The risk of AML rupture is associated with but not exclusive to lesion size. Any decision to intervene AML must be based on multiple factors including risk, symptoms, and auxiliary findings.

## 1 Introduction

Angiomyolipoma (AML) is the most common benign mesenchymal neoplasm of the kidney. The incidence of AML in the general population is estimated to be 0.13%, and it is more prevalent in women than in men.^[1,2]^ AML is often comorbid with tuberous sclerosis complex (TSC), an autosomal dominant disease characterized by benign neoplasms involving multiple systems.^[3]^ The majority of patients with TSC (80%) can develop AML, and TSC is caused by mutations in TSC1 (9q34) or TSC2 (16p13) genes. Similar genetic alteration is also observed in patients with lymphangioleiomyomatosis (LAM).^[4]^ Pathologically AML is caused by clonal proliferation of perivascular epithelioid cells predominantly comprised of fat cells, immature smooth muscle cells, and blood vessels; it is currently thought to belong to a family of tumor collectively referred to as perivascular epithelioid cell tumors. According to the histological composition, there are 2 types of renal AML, a classic type and an epithelioid variant. Specially, epithelioid AML is composed of monotypic epithelial cells and possesses malignant and metastatic potential.

Most AML patients are usually asymptomatic and the diagnosis of AML is often incidental. The classical triad of symptoms characterized by flank pain, palpable mass, and hematuria appeared in less than half of the individuals.^[5,6]^ The main complication of AML is retroperitoneal hemorrhage caused by tumor rupture, which can be severe and life threatening. Acute bleeding of AML manifest as Lenk's triad, including acute flank pain, abdominal tenderness, and signs of internal bleeding such as hematuria; this urological emergency is also called Wünderlich syndrome.^[7,8]^ Currently, the risk of tumor rupture is usually determined by tumor size and surgical approach is necessary for tumor size >4 to 8 cm.^[9,10]^ However, these criteria have been challenged by a series of clinical cases and cohort studies. More importantly, few studies up to date have comprehensively reviewed the risk factors correlated to the rupture and prognosis of AML. Thus, the present paper aimed to review the radiological classification of AML subtypes and provide recent updates of clinical findings associated with AML rupture; by doing so we hope to remind urologists the conventional criteria based on tumor size for AML prognosis may not always be reliable.

## 2 Methods

First, the search term “angiomyolipoma” and “AML” were searched in PubMed and Web of Science, respectively. To avoid ambiguities, MeSH database was used for PubMed search. Then, the “English and Humans” filter was applied to the search results and a total of 1535 articles were found, among which 47 articles significantly related to our topic were included. Additional searches were conducted when any relevant references were discovered in these articles. Finally, 56 articles were reviewed in detail.

The ethic approval was not necessary in this study because no human data or animal experiments were involved.

## 3 Risk factors for AML rupture

The main complication of AML is retroperitoneal hemorrhage caused by AML rupture, which can be severe and lead to a poor prognosis. Risk factors for AML rupture used to focus on tumor size. It has been commonly proposed that tumor with diameter >4 cm is more likely to develop aneurysm and rupture.^[9,10]^ However, a series of clinical studies have reported that hemorrhage and aneurysm formation was not present even in patients with AML >4 cm or with intratumoral aneurysms >5 mm; by contrast, tumor as small as <4 cm may also rupture spontaneously. For example, Gomha et al found that large AML (>10 cm) could also remain stable undergoing conservative management.^[11]^ Prischl and Spottl discovered 9.4% of small AML (<4 cm) could also rupture.^[12]^ More importantly, recent evidences suggest that the rupture of AML may be also influenced by other factors regardless of tumor size.

**Figure 1:**
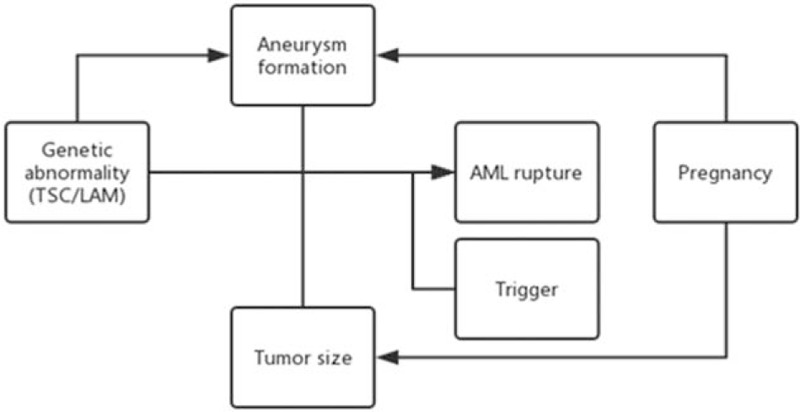
Schematic explanation of AML rupture and its risk factors. Pregnancy and genetic abnormalities contribute to microaneurysm formation and enlarged tumor size, which play the central role in AML rupture. Besides, precipitating factors such as anticoagulation treatment trigger AML rupture. AML = angiomyolipoma.

Considering these challenges to the traditional criteria as well as many cases of ruptured AML, here we propose that risk of AML rupture should be based on a more comprehensive risk assessment system including tumor size, aneurysm formation, pregnancy, coagulopathy, trauma, hormone level, and comorbidity with TSC/LAM. We believe that the interaction among aneurysm formation and TSC/LAM, pregnancy, and the precipitating factors play central role in AML rupture (Fig. 1). Thus, the decision to treat an asymptomatic patient with AML must be also based on this risk assessment system.

### 3.1 Genetic alteration is the innate reason for tumor rupture

Genetic abnormality plays an “initial” role in the complex relationships among all risk factors. It is confirmed that, owing to the uncontrolled mammalian target of rapamycin (mTOR) activation and its angiogenesis effects, TSC patients with loss of *TSC1* or *TSC2* genes were more likely to suffer from aneurysm, which lead to rapid growth and spontaneous rupture of the tumor.^[5]^*TSC1* or *TSC2* mutant genes lead to hyper-activation of mTOR in the regulating pathways in developing fetus, and the disruption of normal angiogenic pathways through hyperactive mTOR signaling may be the mechanism that lead to deranged vascular pathogenesis in the TSC, explaining the tendency of aneurysm formation in TSC individuals.^[13,14]^ Harrington et al^[15]^ found that a major form of negative feedback inhibition of PI3K resulted from activated growth signaling via mTOR and the p70 S6 kinase (S6K), which is responsible for the development of TSC. In addition, other biochemical mechanisms apart from PI3K, such as PAK2, as well as crosstalk between downstream molecules of mTOR may also aggravate the symptoms of AML and induce hemorrhage.^[16,17]^

Because LAM shares the similar genetic characteristics with TSC, patients with LAM were also at an increased risk of AML rupture. By contrast, sporadic AML rarely develop aneurysm or rupture.^[18]^ Based on these findings, TSC and LAM significantly increased the risk of aneurysm formation and AML rupture.

### 3.2 Aneurysm formation influences tumor rupture

Due to the hypervascularity of aneurysms, risk of tumor rupture in AML is related to the presence and size of intratumoral aneurysms, the latter having the strongest association with future risk of rupture. Clinically, image findings described as “aneurysm” are divided into 2 types of pathologic conditions: primary aneurysm and pseudoaneurysm. The primary aneurysms are a “true aneurysm” involving all 3 layers of arterial wall, which can lead to life-threatening retroperitoneal hemorrhage.^[19]^ Another type of “aneurysm,” pseudoaneurysm, is actually a hematoma restricted by surrounding tissues, and is often caused by traumatic events. Pseudoaneurysms are formed by arteries or arterioles lesion and can cause retroperitoneal hemorrhage and even hypovolemic shock after a secondary rupture.^[19,20]^

It has been reported that the aneurysm size >5 mm is more strongly correlated with rupture than tumor size > 4 mm. Using an aneurysm size threshold of 5 mm yields a sensitivity of 100% and specificity of 86% for prediction of hemorrhage. By contrast, tumor size threshold of 4 cm for prediction of future hemorrhage presents a lower statistic value with the sensitivity of 100% and specificity of 38%.^[21]^ Apart from aneurysm size, the proportion of angiogenic component in the tumor may also play an important role in rupture.^[22]^ These data are consistent with the findings of Rimon et al in which they further confirmed that large AML with minimal vascularity tend not to bleed.^[23]^

### 3.3 Pregnancy plays an important role in tumor growth and rupture

In the last 5 years, AML in pregnant individuals has aroused increasing concerns. AML during pregnancy is characterized by faster growth, potentially invasive behaviors, and an increased risk of rupture with massive retroperitoneal hemorrhage.^[24,25]^ In particular, spontaneous renal hemorrhage during pregnancy is rare but the consequences may be catastrophic, including maternal shock and intrauterine fetal death. Pregnancy exerts its negative impacts on AML in many ways. First, the increased number of estrogen and progesterone receptors on smooth muscle cells during pregnancy leads to the deceleration of ureter movement; positive receptors for estrogen and progesterone have been found in more than 25% of these cases. Second, the dilated ureter and enlarged uterine may cause slight hydronephrosis. Besides, the increased blood volume and renal plasma flow during the whole term of pregnancy may disturb the hemodynamics and facilitate aneurysm formation.^[26]^ Finally, the increased blood pressure during 24 to 26 weeks of gestation pregnancy may also contribute to aneurysm formation and rupture directly.

Particularly, a series of pathophysiological changes in AML patients during childbirth including increased muscle sensitivity to oxytocin, enhanced abdominal pressure with uterine contraction, and unstable hemodynamics during delivery also lead to tumor rupture easily. Thus, considering the severe complication of AML rupture during pregnancy and childbirth, it has been suggested that women with known AML who intend pregnancy should be treated prophylactically if the tumor is more than 4 cm, even when asymptomatic, to avoid the risk of rupture.^[27]^

### 3.4 Many precipitating factors triggers AML rupture

The precipitating factors of tumor rupture are various in clinical practice. Snow et al^[28]^ reported a renal AML case with life-threatening rupture and demonstrated that there is a potentially life-threatening association between horse chestnut seed extract-containing products and renal AML. In addition, intracapsular or retroperitoneal hemorrhage of renal AML may result from trauma, even blunt and low-velocity force. In particular, Zengin et al even reported a ruptured renal AML patient caused by hard abdominal physical examination.^[8,29]^

## 4 Classification of renal AML and imaging findings

AML is usually easy to diagnose with imaging alone. It is very important to make precise diagnosis and clinical evaluation of this disease, considering the serious potential complication of retroperitoneal hemorrhage after tumor rupture. Fortunately, the unique features on ultrasonography (US), computed tomography (CT), and magnetic resonance imaging (MRI) usually make AML easily differentiated from other renal masses. The most crucial aims of image checks are to identify and assess those signs associated with tumor rupture such as aneurysm or pseudoaneurysm formation. Therefore, radiologist and clinicians should always know what image features to expect prior to choosing the imaging techniques.

According to histopathology features, AML is classified into potentially malignant epithelioid AML and triphasic benign AML; the latter is further divided into classic AML and fat-poor AML. Classic AML is composed of 3 components: vessels, spindle cells, and adipose tissues. By contrast, fat-poor AML is defined by <25% fat per high power field under microscopy and does not contains enough fat to be detected with images.^[30]^ Here, Jinzaki et al summarized and described the imaging features of renal AML (Table 1).^[31,32]^

**Table 1:**
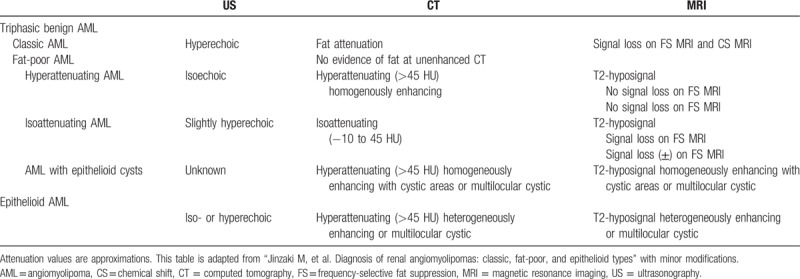
Imaging features of renal angiomyolipoma subtypes.

### 4.1 Ultrasonography

US is a common method to screen AML. Typical appearance of AML on US is a hyperechoic renal lesion with acoustic shadowing. However, owing to the mechanism of imaging, US cannot clearly define AMLs with minimal fat component.^[33]^ Moreover, isoechoic or hyperechoic evidence is often displayed in both fat-poor AML and epithelioid AML. Thus, US is not very sensitive and accurate for differential diagnosis and AML subtype identification.

Contrast-enhanced US can identify active bleeding or pseudoaneurysm formation, and is a valuable real-time diagnostic workup for abdominal emergency caused by AML.^[34]^ Besides that, using color-flow Doppler sonography, the blood flow of tumor can be measured, and solid tumor, aneurysm, and pseudoaneurysm can be distinguished.^[35]^ Therefore, despite the lack of accuracy, US has certain clinical value in the diagnosis of AML.

### 4.2 Computed tomography

CT is the most commonly used method for diagnosing AML. On CT, classical AML appears as predominantly fatty attenuation with various density, whereas fat poor AML is iso- or hyperattenuating with homogeneous enhancing. Epithelioid AML displays a hyperattenuating image with heterogeneous enhancing or multilocular cystic appearance.

Although CT appearance of AML seems distinct, there are several problems that should be noticed by clinicians and radiologists: CT has limited capacity for the detection of minimal AML (nude < 1 cm) because of inaccurate placement of region of interest measurements and volume averaging of voxels containing renal parenchyma and fat; CT imaging is helpful to definitively characterize large AML, but large AML should be differentiated from other fat-containing retroperitoneal masses, such as liposarcoma. Although there are multiple of CT imaging features for describing AML with minimal fat component, most of these findings are overlapped with renal cell carcinoma; More importantly, although CT evidences including thresholds of 4 cm for tumor size and 5 mm for size of aneurysm have been proposed for prediction of future bleeding, the decision to intervene remains controversial as many AML >4 cm or with intratumoral aneurysms >5 mm do not undergo hemorrhage and remain asymptomatic. Therefore, it has been advised that the decision to treat an asymptomatic patient with AML must be based on multiple factors not exclusive to lesion size.^[21]^

Limitations of CT include relatively poor imaging of small vessels. However, digital subtraction angiography (DSA) can display the vascularity of AML clearly.^[36]^ Using this technique, Rimon et al established a scoring system based on CT and DSA appearance to evaluate the hemorrhage risk and to analyze the correlation between hemorrhage and vascularity grading. Their results suggest large AML with minimal vascularity are less likely to bleed, and prophylactic treatment is not necessary.^[23]^

### 4.3 Magnetic resonance imaging

MRI possesses high sensitivity for detecting fat tissues; thus classical AML can be easily identified by MRI. The T1-weighed imaging of MRI displays fat as hyperintense. Comparing the signal intensity between fat-suppressed and nonfat-suppressed sequences helps to further identify the fatty component. On both frequency selective MRI and chemical shift (CS) MRI, classical AML is present as loss of signal with fat suppression which indicates the presence of fat cells. Fat-poor AML is relatively T2-hyposignal, and epithelioid AML is T2-hyposignal with heterogeneously enhancing or multilocular cysts.

It has been established that MRI is useful to differentiate AML from the other renal mass. For example, using the opposed-phase CS-MRI, Israel et al have successfully differentiated renal AML from solid renal masses and hemorrhagic-proteinaceous renal cysts. Their findings suggest “India ink artifact” signal within the renal mass or at the mass–kidney interface is indicative of AML with high sensitivity (100%) and specificity (97.9%).^[37]^ However, compared with DSA and Doppler sonography, MRI has limited ability to outline the vascularity of tumor. Consequently, combination of different imaging techniques is necessary for a comprehensive evaluation of AML.

## 5 Management

Management of AML patients must be based on clear diagnosis for AML and risk assessment for tumor rupture. As previously discussed, diagnostic imaging is of definite value. On the other hand, routine check for AML patients, such as hematology, urine test, coagulation, biochemistry, and hormone level, is mandatory, because these factors may predispose patients to higher risk of tumor rupture. In addition, although biopsy for AML possesses unique immunohistochemical staining and is definitive in the diagnosis of AML, it is rarely used for the risk assessment of tumor rupture clinically.^[5]^

Most AMLs do not require treatment because they are benign and asymptomatic. Indications for treatment include suspicion of malignancy, spontaneous hemorrhage causing significant symptoms, risk of rupture, or other complications. Here Jinzaki et al proposed a treatment scheme for AML of different radiologic class (Table 2).

**Table 2:**
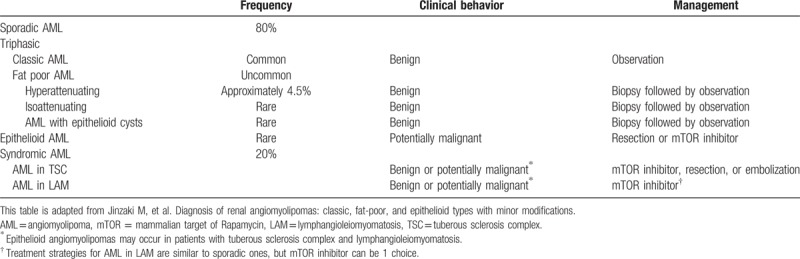
Management of different subtypes of AML based on radiologic findings.

### 5.1 Conservation

Conservation is suitable for asymptomatic lesions <4 cm or some larger but stable masses. Albi et al believed that even Wünderlich syndrome can be also managed conservatively if the hemorrhage is limited and respond to fluid resuscitation^[38]^; however, repeated bleeding may present if the lesion cannot reach complete morphological recovery.^[7]^ Consequently, repeat yearly or semiyearly CT or US can be performed to evaluate the risk of rupture, and those who choose such conservative management should probably avoid contact activities.

### 5.2 Medication

As described in the previous paragraphs, the pathological changes underlying AML rupture is the rapid growth vascularization of the lesion site, and the mTOR plays the central role in the biochemical process of tumor growth and vascularization by acting as a kinase. Recently, mTOR inhibitors (a group of immunosuppressive agents) such as rapamycin and everolimus have been proved to be effective for AML patients by inhibiting vascular epithelial proliferation and reducing the tumor size.^[39]^ Moreover, this therapy can also reduce the risk of rupture and bleeding.^[40]^

Because patients with TSC or LAM have genetic abnormalities in *TSC1* or *TSC2* genes and thus uncontrolled mTOR activation, medication can be especially beneficial to patients with these patients. Therefore, literatures have recommended mTOR inhibitors for TSC/LAM patients to control and reduce tumor size.^[41]^

### 5.3 Embolization

Controversies over the choice between embolization and surgery mainly focus on the safety, efficacy, renal preserve, and pregnancy. Although both surgery and embolization are useful for patients with AML <4 cm, currently renal artery embolization is recommended as a first-line therapy for bleeding AML and is increasingly used as a preventive treatment for AML at risk of bleeding.^[42]^ Moreover, compared with surgical alternatives, embolization possesses several advantages including a low complication rate, less trauma,^[43]^ renal function preserve,^[20]^ and satisfactory short-term (<5 years) outcome.^[44,45]^ Ewalt et al found that transcatheter embolization of large AML prevented hemorrhage and renal loss; this intervention is minimally invasive and preserves renal function.^[46]^ Wang et al, respectively, reviewed 46 patients who underwent super-selective renal artery embolization (SRAE) for renal hemorrhage, and indicated that SRAE is an effective and minimally invasive method for the control of renal hemorrhage.^[47]^ Choices of embolization agents and methods significantly determine the outcome of embolization and should be guided by the hemodynamics of patients.^[48]^ Development and application of new embolization agents may help interventionist better cope with clinical practice.^[49]^

Although embolization is the currently preferred treatment of symptomatic or ruptured renal AML with high safety and well renal preserve, limitations of embolization also exist. First, it should be noted that although embolization induced tumor shrinkage in most patients, tumor shrinkage alone is not a reliable exclusion criterion for recurrent hemorrhage^[50,51]^; Boorjian et al pointed out that embolization had higher risk of relapse and recurrent bleeding compared with partial nephrectomy.^[52]^ Second, it is argued that selective embolization was not devoid of complications; Kara et al recommended RPN as an option for patients with increased risk of rupture.^[53]^

Embolization has limited application in several scenarios: multiple or giant aneurysm, as seen in TSC and LAM respond poorly to embolization; large ratio of fat to vascularity always indicate poor outcome of the procedure^[50,54]^; large proportion of vascularity may also require repeated embolization due to the complexity of vascular arrangement^[51]^; and owing to the radioactive hazard during embolization, it is not applicable to pregnant patients who wish to preserve the fetus.

### 5.4 Surgery

Surgery was the classical treatment for symptomatic AMLs until the recent emergence of embolization techniques. Nevertheless, surgery still remained an important option for large, symptomatic AMLs. Nephron-sparing surgery (NSS) is now widely accepted as the optimal operation in clinical practice for its feasibility, efficacy, and satisfactory renal preserve. In a retrospective study conducted by Yip et al, they considered that NSS is feasible and effective for renal AML, even for massive AML or after previous rupture, especially when the diagnosis was made by preoperative imaging and/or intraoperative frozen section.^[55]^ Boorjian et al reviewed patients undergoing NSS from 1970 to 2004 in their institution and concluded NSS offered preservation of renal function and was associated with acceptable complication (12% rate of complication, including 5% urine leakage) and low local recurrence rates.^[52]^ Furthermore, recent development of robotic technology allows surgeons to improve operation outcome by decreasing complication rate and increasing renal preserve.^[53]^ In conclusion, it is believed that surgery will continue to play important roles in the treatment of AML.

### 5.5 Treatment for pregnant patients

As discussed, pregnancy is also a contraindication of embolization. Due to the increased risk of tumor rupture and invasion, termination of pregnancy should be considered in patients diagnosed with AML during early pregnancy.^[56]^

It has been advised that women with known AML who intend pregnancy should be treated prophylactically if the tumor is more than 4 cm, even when asymptomatic, to avoid the risk of rupture. The patient will for delivery, gestational week, fetal and mother's state should also be taken into consideration. Surgery is a better choice for full-term pregnancy women with AML as it can manage the renal condition without compromising the safety of mother and fetus; obstetric emergencies, such as fetal distress, dystocia, placenta praevia, are strong indicators for joint surgery of nephrectomy and caesarean delivery. Nephrectomy yielded good prognosis for both mother and fetus in many cases and has proved to be successful in AML during pregnancy.^[26,57,58]^ Although there are some case reports that multidisciplinary approach may leave the fetus in gestation whilst facilitating radical nephrectomy,^[59]^ management of such cases still remains challenging.

## 6 Conclusion

Renal AML is a common benign tumor composed of dysmorphic blood vessels, smooth muscle, and mature adipose tissue with varying proportions. The main complication of AML is retroperitoneal hemorrhage caused by tumor rupture, which can be severe and life threatening. The clinical intervention of AML is mainly based on classification of the tumor and the risk of AML rupture. It has been widely accepted that AML tumor size >4 cm or intratumoral aneurysms >5 mm is the recommended criteria to determine the risk of tumor rupture. However, these criteria are not always reliable, as many patients with AML >4 cm or with aneurysms >5 mm did not undergo rupture or aneurysms formation. Thus, the current criteria are hardly satisfactory. The authors believe that prediction of AML rupture should be assessed on a more comprehensive risk assessment system including epidemiologic, pathologic features, and imaging evidence. The treatment strategy should be compatible with the risk factors discussed above, because patients with different risk factors showed different tolerance and prognosis. Unfortunately none of the current studies have managed to cover all risk factors. The paucity of literatures available on the prognostic factors of AML also set limit to our review. As more clinical researches and data emerges, it should be expected that a detailed scoring system can be established to help identify patients in need for prophylactic intervention.

## Author contributions

**Formal analysis:** Xin Gou.

**Investigation:** Jing Fan.

**Methodology:** Chenyang Wang.

**Software:** Linglong Peng.

**Writing – original draft:** Xinyuan Li.
